# Rapid Drop-Volume Electrochemical Detection of the “Date Rape” Drug Flunitrazepam in Spirits Using a Screen-Printed Sensor in a Dry-Reagent Format

**DOI:** 10.3390/s20185192

**Published:** 2020-09-11

**Authors:** Frixos Papadopoulos, Konstantinos Diamanteas, Anastasios Economou, Christos Kokkinos

**Affiliations:** Department of Chemistry, National and Kapodistrian University of Athens, 157 71 Athens, Greece; frixospapp13@yahoo.gr (F.P.); diamanteaskonstantinos@hotmail.com (K.D.); christok@chem.uoa.gr (C.K.)

**Keywords:** flunitrazepam, cyclic voltammetry, screen-printed sensor, date-rape drugs, alcoholic drinks

## Abstract

Flunitrazepam is an extremely potent benzodiazepine sedative which is associated with “drug-facilitated sexual assault” when administered within an alcoholic drink. This work describes a simple electrochemical method for on-site rapid detection of flunitrazepam in untreated spirits (whiskey, vodka and gin) using a single-use screen-printed sensor (featuring graphite working and auxiliary electrodes and an Ag/AgCl reference electrode) in a dry reagent format. Analysis was performed by placing a drop of sample on the sensor, which was previously coated with dry KCl, and recording selected reduction/oxidation peaks of the target compound in a cyclic voltammetry scan. The limit of quantification of flunitrazepam was at the sub-mg L^−1^ range. The between-sensor % relative standard deviation of the analytically useful reduction peak in a solution containing 11.4 mg L^−1^ flunitrazepam was 9.8% (*n* = 5). Quantification was performed using calibration curves constructed from pooled samples spiked with flunitrazepam with relative errors <15%. The main advantages of the methodology are that it involves no sample pretreatment (such as deoxygenation, extraction or reagent(s) addition) and requires only drop-sized volumes of the sample, thus facilitating rapid on-site screening using portable equipment.

## 1. Introduction

Flunitrazepam (6-(2-fluorophenyl)-2-methyl-9-nitro-2,5-diazabicyclo [5,4,0]ndeca-5,8,10,12-tetraene-3-one) is a benzodiazepine first synthesized in 1972 by Roche as a potent sedative to treat severe insomnia [1,2]. Ιt is legally prescribed in more than 50 countries in Europe, Africa, Latin America, Middle East, Asia, and Australia but also is illegally found in countries in which its medical use is not approved, notably in the United States [1]. Flunitrazepam is known by different trade names (Rohypnol^®^, Roipnol^®^, Fluninoc^®^, Silece^®^, Hipnosedon^®^, Nervocuril^®^ etc.) and colloquially known as “roofies”, “the forget pill”, “La Rocha” and “Mexican Valium” [3]. Flunitrazepam is used as a recreational drug popular in clubs and rave parties and believed to be the most commonly used “date-rape drug” associated with “drug-facilitated sexual assault” [1,2,4,5]. In view of the increasing number of “drug-facilitated sexual assault” cases, national and international bodies have expressed their concern: the World Health Organization identifies the implicative role of “date-rape” drugs in incidents of sexual violence [6]; the Parliamentary Assembly of the Council of Europe took steps in order to raise the public’s awareness to sexual assaults linked to “date-rape” drugs [7]; and, the U.S. Congress passed the Drug-Induced Rape Prevention and Punishment Act, which imposes stiff sentences for “drug-facilitated sexual assault” crimes and for the importation and distribution of flunitrazepam [8,9]. In a typical case of “drug-facilitated sexual assault”, flunitrazepam is administered to an alcoholic beverage of an unsuspecting victim; the clinical manifestations of the drug, compounded by consumption of ethanol, include drowsiness, impaired psychomotor activity, confusion, ataxia, paradoxical reaction, and anterograde amnesia [10]. Flunitrazepam is colorless, odorless, and tasteless and is easily dissolved in drinks; the recommended pharmacological dose is around 1 mg in adults, but, when used as a “date-rape” drug, it is usually spiked into alcoholic drinks at higher doses (at around 2 mg it causes mild impairment and at >5 mg it induces strong sedation and amnesia). Once ingested, its action begins after 20–30 min, peaks at 2 h while residual effects can persist up to 12 h or more [2,11]. Since the drug is rapidly metabolized and excreted from the body, its determination in body fluids becomes challenging [1,2,11].

Many analytical methods have been reviewed in literature for the determination of benzodiazepines (including flunitrazepam) in different matrices [12]. Benzodiazepines are electrochemical active (possessing a reducible azomethine group) and can be electrochemically determined by a host of dynamic electroanalytical techniques, as well as potentiometry [13]. In particular, voltammetric techniques, in combination with screen-printed sensors, enable on-site screening tests which are rapid, cost-effective, and can be realized without sample pre-treatment using portable equipment [14]. Garcia-Gutierrez and Lledo-Fernandez [15] and Smith et al. [16] have reported the voltammetric determination of flunitrazepam in drinks using graphite screen-printed electrodes; however, these methods were applicable to low alcohol content (<5% *v*/*v*) beverage samples with deoxygenation and involved immersion of the sensor in the sample. More recently, Tseliou et al. have proposed a voltammetric method for the drop-volume determination of flunitrazepam in alcoholic and non-alcoholic drinks using a graphite screen-printed sensor sparked with Fe nanoparticles (to enhance sensitivity) and modified with glucose oxidase and glucose (to alleviate the oxygen interference) [17]; however, the relative complexity of the sensor’s fabrication process and the use of an expensive enzyme limits its utility for routine analysis. In this work, we propose a method for the rapid electrochemical detection of flunitrazepam in spirits (whiskey, vodka, and gin). Spirits are a common means for administering the drug for criminal purposes since the drink is rapidly consumed in a single “shot” while the high alcohol content amplifies the symptoms. The method utilizes a plain graphite screen-printed sensor in a dry reagent format and is directly applicable to the drop-volume analysis of spirits without pre-treatment (such as deoxygenation or extraction) using portable instrumentation.

## 2. Materials and Methods

### 2.1. Chemical and Reagents

All the chemicals were of analytical grade and purchased from Merck (Darmstadt, Germany) or Sigma-Aldrich (St. Louis, MO, USA). Ketamine, flunitrazepam and gamma-hydroxy butyric acid lactone (GBL) were from Sigma-Aldrich; gamma-hydroxybutyric acid (GHB) sodium salt was prepared and characterized as described previously [18]. Doubly distilled water was used throughout. A stock solution containing 240 mg L^−1^ of flunitrazepam was prepared in doubly distilled water. Screen-printed sensors (DRP 11 L featuring carbon working and counter electrodes and Ag/AgCl reference electrode) were purchased from DropSens (Oviedo, Spain).

Five samples of whiskey (including 2 single-malt, 2 mixed–malt and 1 bourbon brands), as well as 5 samples of each gin and vodka from different brands, were purchased from a local store. A pooled sample of each beverage was prepared by mixing equal volumes of the 5 individual samples; diluted pooled samples were also prepared by dilution of the pooled sample 1:1 (*v*/*v*) and 1:4 (*v*/*v*) with water. Calibration plots for flunitrazepam were constructed by spiking the pooled samples and diluted pooled samples with the appropriate amounts of the flunitrazepam stock solution.

### 2.2. Instrumentation

Electrochemical experiments were performed with a Palmsens potentiostat controlled by the PSTrace 4.2 software (Palm Sens BV, The Netherlands) installed in a laptop computer. The potentiostat was connected to the screen-printed sensor using a PalmSens SPE connector.

### 2.3. Experimental Procedure

**Figure 1:**
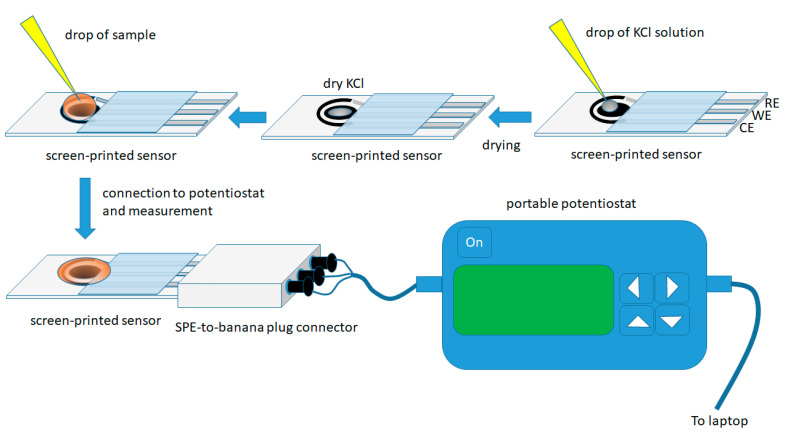
Experimental procedure for the determination of flunitrazepam in alcoholic beverages using the screen-printed sensor in the dry-reagent format.

The experimental procedure is illustrated in Figure 1.

Screen-printed sensors were modified in the lab by placing a 1 µL drop of a 4.0 mol L^−1^ KCl solution on the working electrode and left to dry at room temperature. These sensors modified with dry KCl are stable for at least a month and can be directly applied to on-site assays without further addition of reagent(s) in the sample.

For the analysis, a 50 µL drop of the sample was placed on the working area of the sensor (in order to cover the three electrodes) and, after 30 s (to allow the dry KCl to dissolve), a cyclic voltammogram (CV) was recorded in the potential range +0.50 V to −1.50 V (with respect to the Ag/AgCl quasi-reference electrode) at a scan rate of 50 mV s^−1^; three consecutive CV scans were recorded in each sample and all the measurements were recorded in non-deoxygenated solutions. A new electrode was used for each measurement.

## 3. Results and Discussion

### 3.1. Method Development

The aim of this work was to develop a simple, cost-effective, and rapid test for the target compound in spirits with minimal sample pretreatment.

Regarding the electrochemical transducer element, a commercially available screen-printed sensor, featuring carbon working and counter electrodes and an Ag/AgCl reference electrode, was chosen. The sensor can be easily connected to portable instrumentation (Figure 1), thus enabling on-site analysis and is disposable minimizing the risk of carry-over effects between samples.

**Figure 2:**
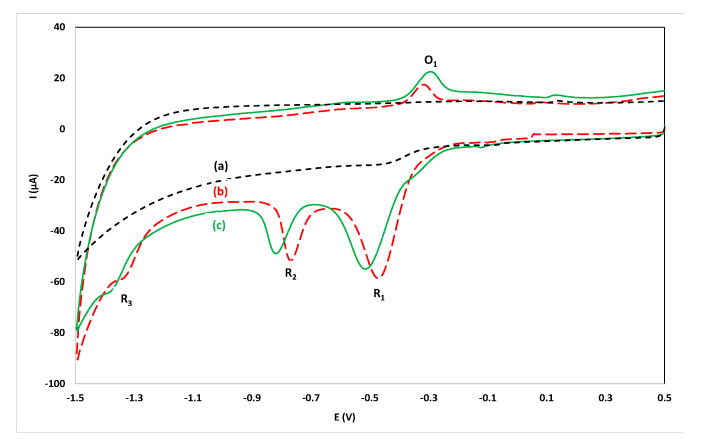
Cyclic voltammograms in the range 0.50 V to −1.50 V: (**a**) immersing the sensor in an aqueous solution of 0.08 mol L^−1^ KCl; (**b**) as (**a**) after addition of 15.7 mg L^−1^ flunitrazepam; (**c**) at a 50 µL drop of an aqueous solution containing 15.7 mg L^−1^ flunitrazepam at the sensor modified with dry KCl (1 µL drop of 4.0 mol L^−1^ KCl).

In previous work on the voltammetric determination of flunitrazepam, phosphate buffer (in the pH range 2.0–7.0) has been used as supporting electrolyte [15,16,17]. Since it is desirable to operate in a reagentless mode, initial experiments were conducted by recording CVs by direct immersion of the sensor in alcoholic drinks without the addition of supporting electrolyte but the conductivity of these solutions was poor. The conductivity was restored, and satisfactory CVs were recorded, after spiking the samples with KCl (200 µL of a 4.0 mol L^−1^ KCl solution in 10 mL of sample). KCl was additionally selected as the supporting electrode in order to provide a constant Cl^-^ concentration to stabilize the potential of the Ag/AgCl quasi-reference electrode. A CV of an aqueous solution containing 15.7 mg L^−1^ flunitrazepam in 0.08 mol L^−1^ KCl is illustrated in Figure 2b. The redox mechanism of flunitrazepam has been studied earlier [15,16,17]: the reduction peak R_2_ arises from reduction of the 7-nitro group to hydroxylamine, the reduction peak R_3_ is due to reduction of the hydroxylamine group to the respective amine, the oxidation peak O_1_ is likely to result from the oxidation of the hydroxylamine to the nitroso analogue which is reduced back to hydroxylamine (peak R_1_). It was observed that the reduction peak R_1_ was not reproducible while the reduction peak R_3_ was hardly observed as it coincided with the hydrogen evolution wave. For analytical purposes, the reduction peak R_2_ and the oxidation peak O_1_ could be used successfully.

In order to simplify the analytical protocol and convert it into a dry reagent drop-volume format, the sensor was modified with dry KCl (by placing a 1 µL drop of 4.0 mol L^−1^ KCl solution on the working electrode of the screen-printed sensor and leaving to dry). Then a 50 µL drop of the sample was placed on the working area of the sensor and the CV was recorded. A CV taken using the dry reagent drop-volume format using a 50 µL drop of an aqueous solution containing 15.7 mg L^−1^ flunitrazepam on the sensor modified with dry KCl is illustrated in Figure 2c. It is clear that well-defined peaks were obtained using the dry-reagent format only exhibiting a potential shift with respect to the solution-phase format in Figure 2b (which is attributed to incomplete dissolution of the dry KCl in the time-scale of the experiment). In this way, several sensors can be easily prepared with the dry reagent, alleviating the need for reagent addition in the sample solution, hence simplifying the analytical procedure.

It is a further critical aspect of the procedure that, under the conditions of the analysis, oxygen did not interfere with the detection of flunitrazepam and no prior deoxygenation step of the sample was required.

### 3.2. Analytical Features

**Figure 3:**
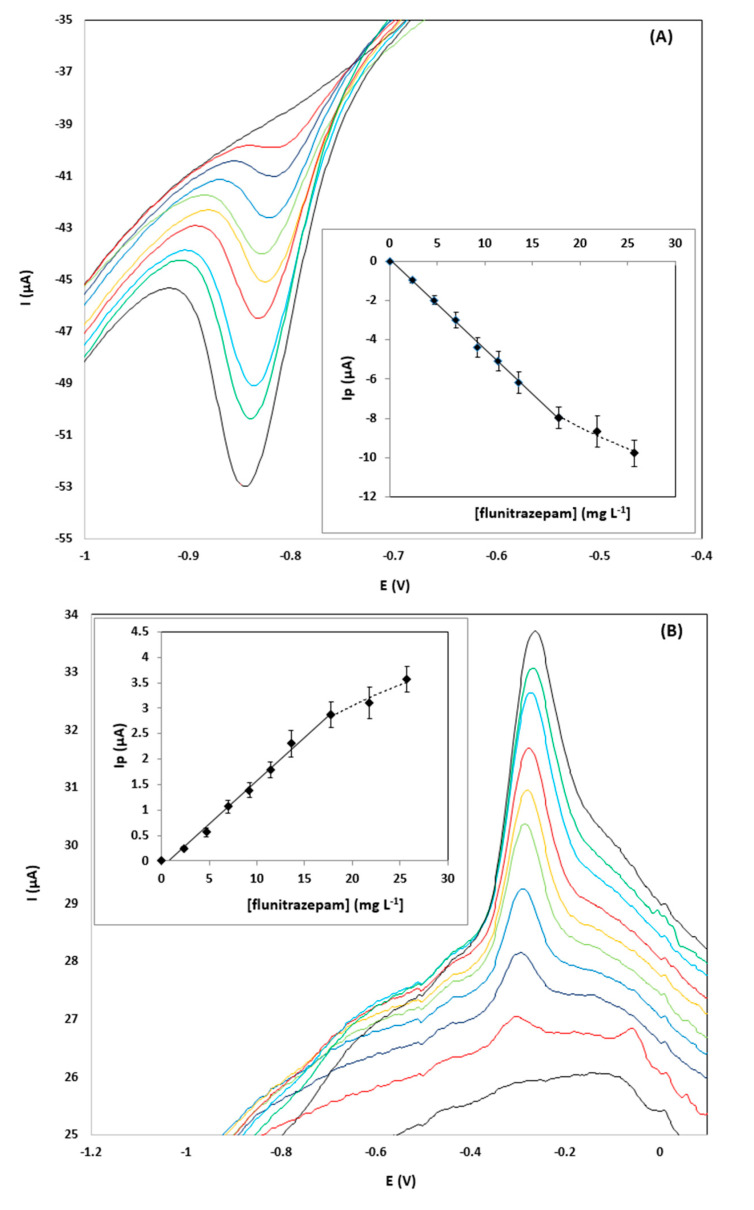
CVs for the determination of 0–25.7 µg L^−1^ of flunitrazepam in a pooled gin sample diluted 1:1 (*v*/*v*) using the drop-volume detection format using: (**A**) the reduction peak R_2,_ and; (**B**) the oxidation peak O_1_.

Calibration of flunitrazepam in the concentration range 0–25.7 mg L^−1^ was performed using the pooled samples and the diluted pool samples spiked with flunitrazepam. The calibration features are summarized in Table 1. Representative CVs showing the reduction peak R_2_ and the oxidation peak O_1,_ together with the respective calibration plots for a gin sample diluted 1:1 (*v*/*v*), are illustrated in Figure 3; the signal-to-noise ratio (S/N) of the reduction peak R_1_ is shown to be markedly better than the S/N ratio of the oxidation peak O_1._

The limits of detection obtained with the proposed sensor were in the range 0.57–4.6 mg L^−1^, depending on the type of sample and the dilution level. To put these data into perspective, to achieve the 1 mg pharmacological dose, a pill containing 1 mg of flunitrazepam should be added in a portion of a spirit (around 40 mL) and the resulting concentration in the drink would be 25 mg L^−1^. This is actually the minimum concentration expected in a typical scenario of “drug-facilitated sexual assault” since flunitrazepam is usually spiked into alcoholic drinks at higher doses than the pharmacological dose. Therefore, the limits of quantitation (LOQs) of the proposed methodology are sufficient for quantifying the minimum concentration of the drug even at 1:4 (*v*/*v*) dilution of the sample (corresponding to 5 mg L^−1^ of flunitrazepam in the solution to be analyzed). The LOQs using the reduction peak R_2_ were generally lower than using the oxidation peak O_1_.

From inspection of the slopes of the calibration plots, it is clear that a significant matrix effect occurred since the slope increased with increasing sample dilution. Statistically significant differences in sensitivity between the pooled whiskey, gin, and vodka samples were also observed. In view of the different slopes in different types of samples and dilutions, quantification issues are further discussed in paragraph 3.4.

The between-sensor reproducibility was estimated by performing measurements of a standard solution containing 11.4 mg L^−1^ of flunitrazepam at five different sensors prepared on the same day; the % relative standard deviations were 9.8% for the reduction peak R_2_ and 11.1% for the oxidation peak O_1_. Considering the LOQs, the S/N ratios and repeatability/reproducibility, the reduction peak R_2_ was selected for quantification in subsequent experiments. The shelf-life of the KCl-modified sensors (i.e., the stability of the KCl-modified sensors under storage in ambient conditions) was assessed by preparing 21 different sensors on the same day and performing 3 consecutive measurements of a standard solution containing 11.4 mg L^−1^ of flunitrazepam every 4 days. As demonstrated in Figure S1 (Supplementary Materials), the response remained statistically stable within the 25 days of the study.

### 3.3. Interference Study

No peaks interfering with the analytical peaks of flunitrazepam were observed in the CVs of the whiskey, vodka and gin samples analyzed, therefore no endogenous redox species in the samples interfered electrochemically with the detection of the target compound.

The interference study included the two other main date-rape drugs [2], gamma-hydroxybutyric acid (GHB) and ketamine, as well as scopolamine which is also well known to be used for predatory purposes [19]. None of these compounds produced peaks in the CV scan or caused any change in the flunitrazepam peak heights (Figure S2, Supplementary Materials). Indeed, GHB is known to be oxidized only under very specific conditions [18] while both ketamine [20] and scopolamine [19] are oxidized at more positive potentials.

### 3.4. Application

**Figure 4:**
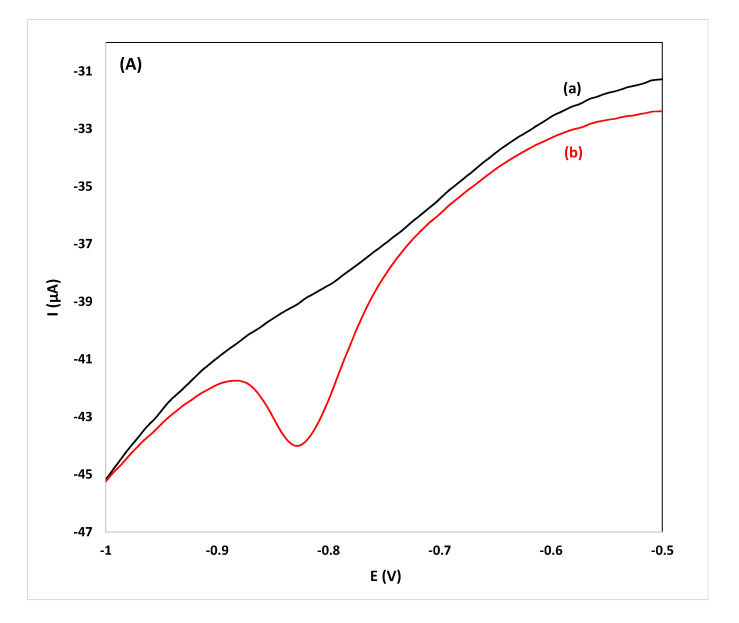

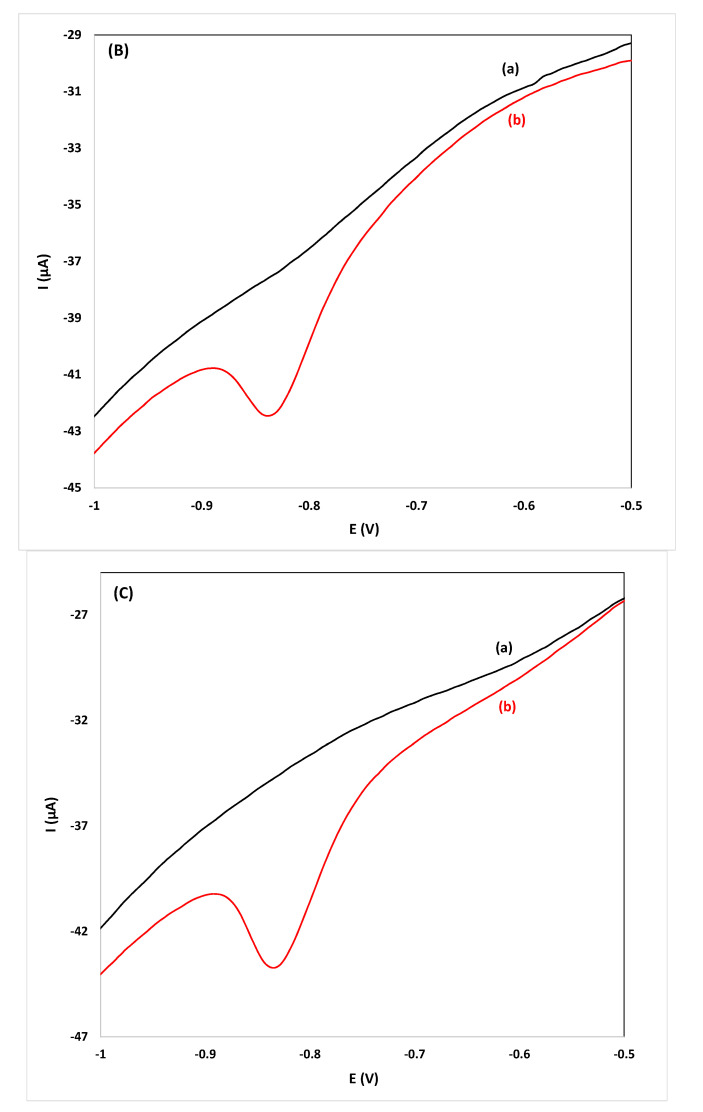
CVs for the determination of flunitrazepam using the reduction peak R_2_ in: (**A**) gin, ((**B**) vodka) and, (**C**) whiskey samples diluted 1:1 (*v*/*v*), (a) before and (b) after spiking with 11.4 mg L^−1^ of flunitrazepam.

While the primary goal is screening for the target compound in spirits, quantification is also possible using the proposed drop-volume methodology. For this purpose, calibration curves were constructed from pooled samples and diluted pooled samples spiked with flunitrazepam and were stored in PSTrace 4.2 operating in the analytical mode. To test the accuracy of the methodology developed in this work, three individual samples each of gin, whiskey and vodka (undiluted and diluted 1:1 (*v*/*v*) and 1:4 (*v*/*v*)) were spiked with flunitrazepam and were analyzed. The concentration was calculated using the respective stored calibration plot constructed using pooled samples or diluted pooled samples using electrodes randomly selected from three batches purchased at different times. The values of the relative errors for all the samples analyzed were <15% and the method was deemed accurate for the intended purpose (Table 2). Quantification using stored calibration curves of pooled samples is faster and more convenient than applying the method of standard additions since only a single measurement per sample is required; the total analysis time (from sample addition to reporting of the result) is less than 90 s. Voltammograms for the detection and quantification of flunitrazepam in individual samples of spirits are illustrated in Figure 4.

## 4. Conclusions

In this work, an electrochemical method is developed for on-site rapid detection of the date-rape drug flunitrazepam in spirits (whiskey, vodka, and gin) using a single-use screen-printed sensor in a dry reagent format. The main advantages of the methodology are that it is extremely simple and fast, involves neither sample pretreatment (such as deoxygenation, extraction or reagent(s) addition) nor electrode modification, requires only drop-sized volumes of the sample (thus facilitating rapid on-site screening using portable equipment) while a single measurement is sufficient for quantitative analysis. The method proved fit-for-purpose and is suitable both for screening purposes and for quantification of the target compound in alcoholic drinks.

## Figures and Tables

**Table 1:** Calibration features in pooled spirit samples using the reduction peak R_2_ and the oxidation peak O_1_.

Sample		Slope (µA mg^−1^ L)	R ^1^	LOQ (mg L^−1^) ^2^
**Reduction peak R_2_**				
Whiskey	No dilution	−0.288 ± 0.023	0.993	0.93 (0.93)
	Diluted 1:1	−0.365 ± 0.011	0.994	1.0 (2.0)
	Diluted 1:4	−0.955 ± 0.024	0.997	0.63 (3.2)
Gin	No dilution	−0.136 ± 0.003	0.996	0.81 (0.81)
	Diluted 1:1	−0.456 ± 0.009	0.997	0.57 (1.1)
	Diluted 1:4	−0.688 ± 0.022	0.993	0.71 (3.6)
Vodka	No dilution	−0.255 ± 0.007	0.996	0.76 (0.76)
	Diluted 1:1	−0.271 ± 0.009	0.993	1.1 (2.2)
	Diluted 1:4	−0.473 ± 0.017	0.990	0.75 (3.8)
**Oxidation peak O_1_**				
Whiskey	No dilution	0.087 ± 0.021	0.989	0.96 (0.96)
	Diluted 1:1	0.143 ± 0.033	0.997	0.67 (1.2)
	Diluted 1:4	0.342 ± 0.009	0.996	0.79 (4.0)
Gin	No dilution	0.075 ± 0.0032	0.986	1.7 (1.7)
	Diluted 1:1	0.169 ± 0.006	0.993	1.0 (2.0)
	Diluted 1:4	0.368 ± 0.013	0.992	1.1 (4.4)
Vodka	No dilution	0.124 ± 0.003	0.995	1.0 (1.0)
	Diluted 1:1	0.134 ± 0.005	0.989	1.5 (3.0)
	Diluted 1:4	0.379 ± 0.014	0.993	0.91 (4.6)

^1^ Coefficient of determination. ^2^ Limit of quantification (in parenthesis the LOQ values in the original sample before dilution).

**Table 2:** Accuracy for the determination of flunitrazepam in individual spirits using the reduction peak R_2_.

Sample		Dilution (*v*/*v*)	Added (mg L^−1^)	Found (mg L^−1^)	E_r_ (%) ^1^
Whiskey	1 ^2^	No	11.4	13.0	14
	2 ^3^	1:1		12.6	10
	3 ^4^	1:4		12.2	6.8
Gin	1	No		10.4	−8.8
	2	1:1		12.1	6.2
	3	1.4		10.8	−5.2
Vodka	1	No		12.8	12
	2	1:1		12.6	10
	3	1:4		11.2	−11

^1^ % error; ^2^ single-malt; ^3^ mixed-malt; ^4^ bourbon.

## Supplementary Materials

The following are available online at https://www.mdpi.com/1424-8220/20/18/5192/s1, Figure S1: Shelf-life test of the sensors, Figure S2: Selectivity of the sensors towards ketamine, GHB and scopolamine.
