# Supplementary material for: Rapid Drop-Volume Electrochemical Detection of the “Date Rape” Drug Flunitrazepam in Spirits Using a Screen-Printed Sensor in a Dry-Reagent Format
Source: Sensors (Basel). 2020 Sep 11;20(18):5192. doi: 10.3390/s20185192 (PMC7570630; doi:10.3390/s20185192)
Supplement: Supplementary file 1 [file sensors-20-05192-s001.pdf]

Supplementary Material

# Rapid drop-volume electrochemical detection of the "date rape" drug flunitrazepam in spirits using a screen-printed sensor in a dry-reagent format

**Frixos Papadopoulos, Konstantinos Diamandeas, Anastasios Economou, Christos Kokkinos**

Department of Chemistry, National and Kapodistrian University of Athens, Athens 157 71, Greece

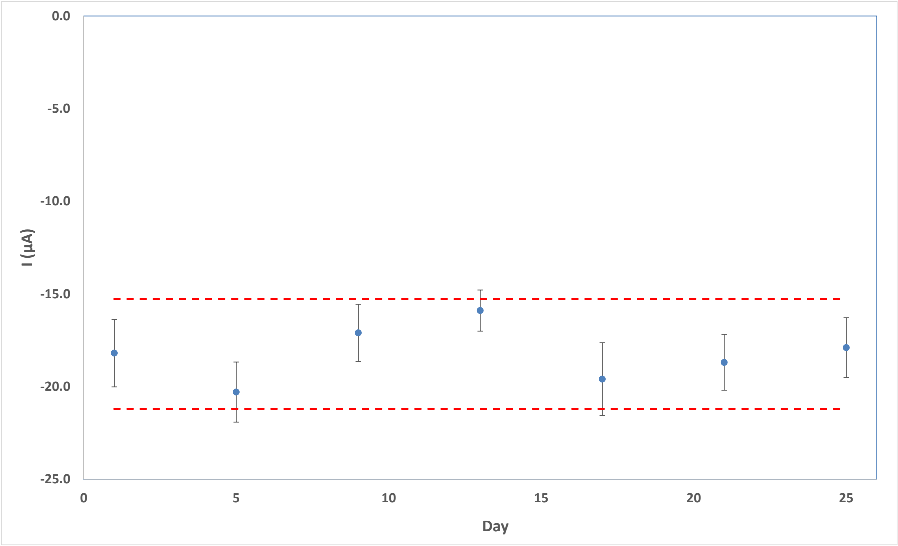


**Figure S1.** Shelf-life test of the sensors for the detection of 11.4 µg $L^{-1}$ of flunitrazepam in a standard solution using the drop-volume detection format and the reduction peak $R_2$. Each point is the average of three measurements performed on the same day. The red dotted lines correspond to the ± 2SD limits (where SD is the standard deviation of the total 21 measurements).

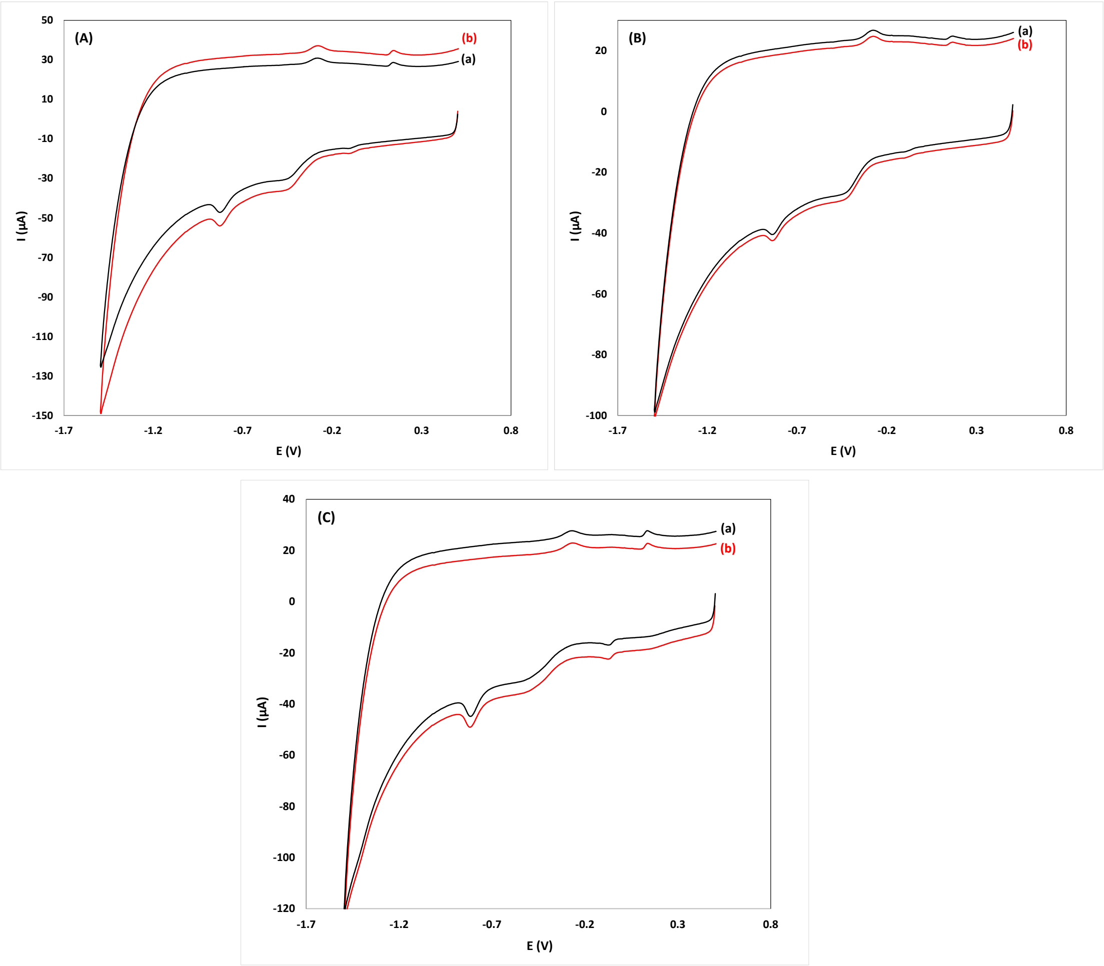


**Figure S2.** CVs in: (A) gin; (b) vodka, and; (C) whiskey pooled samples diluted 1:1 (v/v) and containing: (a) 11.4 mg $L^{-1}$ of flunitrazepam, and; (b) 11.4 mg $L^{-1}$ of each flunitrazepam, ketamine, scopolamine and GHB.
